# Identification of candidate regulators of mandibular bone loss in *FcγRIIB*^-/-^ Mice

**DOI:** 10.1038/s41598-021-98108-3

**Published:** 2021-09-21

**Authors:** Nithidol Sakunrangsit, Jatuphol Pholtaisong, Jeerus Sucharitakul, Sasithorn Wanna-udom, Pinidphon Prombutara, Prapaporn Pisitkun, Asada Leelahavanichkul, Chatchawit Aporntewan, Matthew B. Greenblatt, Sutada Lotinun

**Affiliations:** 1 Skeletal Disorders Research Unit, Department of Physiology, Faculty of Dentistry, Chulalongkorn University, Bangkok, 10330 Thailand; 2 Program in Bioinformatics and Computational Biology, Graduate School, Chulalongkorn University, Bangkok, 10330 Thailand; 3 Department of Biochemistry, Faculty of Dentistry, Chulalongkorn University, Bangkok, 10330 Thailand; 4 Omics Sciences and Bioinformatics Center, Faculty of Science, Chulalongkorn University, Bangkok, 10330 Thailand; 5 Division of Allergy, Immunology, and Rheumatology, Department of Medicine, Faculty of Medicine, Ramathibodi Hospital, Mahidol University, Bangkok, 10400 Thailand; 6 Division of Immunology, Department of Microbiology, Faculty of Medicine, Chulalongkorn University, Bangkok, 10330 Thailand; 7 Department of Mathematics and Computer Science, Faculty of Science, Chulalongkorn University, Bangkok, 10330 Thailand; 8 Department of Pathology and Laboratory Medicine, Weill Cornell Medicine, and Research Division, Hospital for Special Surgery, New York, NY 10065 USA

**Keywords:** RNA sequencing, Bone

## Abstract

Patients with systemic lupus erythematosus (SLE) have increased inflammatory cytokines, leading to periodontitis and alveolar bone loss. However, the mechanisms driving this phenomenon are still unknown. Here, we have identified novel therapeutic targets for and mediators of lupus-mediated bone loss using RNA-sequencing (RNA-seq) in a *FcγRIIB*^-/-^ mouse model of lupus associated osteopenia. A total of 2,710 upregulated and 3,252 downregulated DEGs were identified. The GO and KEGG annotations revealed that osteoclast differentiation, bone mineralization, ossification, and myeloid cell development were downregulated. WikiPathways indicated that Hedgehog, TNFα NF-κB and Notch signaling pathway were also decreased. We identified downregulated targets, *Sufu* and *Serpina12*, that have important roles in bone homeostasis. *Sufu* and *Serpina12* were related to Hedgehog signaling proteins, including Gli1, Gli2, Gli3, Ptch1, and Ptch2. Gene knockdown analysis demonstrated that *Sufu*, and *Serpina12* contributed to osteoclastogenesis and osteoblastogenesis, respectively. Osteoclast and osteoblast marker genes were significantly decreased in *Sufu*-deficient and *Serpina12*-deficient cells, respectively. Our results suggest that alterations in Hedgehog signaling play an important role in the pathogenesis of osteopenia in *FcγRIIB*^-/-^ mice. The novel DEGs and pathways identified in this study provide new insight into the underlying mechanisms of mandibular bone loss during lupus development.

## Introduction

Periodontitis is an oral infectious disease that impacts dental support tissues and destroys periodontal bone. Chronic periodontitis is driven by inflammatory reactions to microorganisms in the dental plaque and impairs osseous coupling, leading to osteoclast-mediated bone resorption and subsequent tooth loss. Patients with chronic periodontitis have high level of RANKL which is correlated with increased number of Gram-negative bacteria, such as *Porphyromonas gingivalis* in periodontal tissue^1^. This organism has virulence factors that induce inflammatory responses, eventually leading to alveolar bone loss. Chronic inflammatory diseases such as diabetes, rheumatoid arthritis, and systemic lupus erythematosus (SLE) are associated with an increased risk of periodontal disease. The prevalence and severity of periodontal diseases, including gingivitis and periodontitis are increased in patients with SLE.

SLE is a multisystem autoimmune disorder that produces autoantibodies resulting in extensive inflammation and tissue destruction. SLE patients have a higher prevalence of periodontal disease due to dysbiotic subgingival microbiota with a shift toward pathogenic bacteria^2^. However, the mechanism by which SLE induces mandibular alveolar bone loss has not been identified. Fc gamma receptor (FcγR) is essential for many immune system effector functions, including phagocytosis, antibody-dependent cellular cytotoxicity and inflammation. Most FcγRs identified in mice, FcγRI, FcγRIII, FcγRIV, are activating receptors which transduce signals through the phosphorylation of immunoreceptor tyrosine-based activating motifs (ITAM)^3^. The only inhibitory FcγR, FcγRIIB, is a key player in regulating autoimmunity and inflammatory response to immune complexes. *FcγRIIB* is expressed in B cells, granulocytes, dendritic cells, and macrophages^3^. Mice lacking *FcγRIIB* exhibit an exacerbated immune response, and are prone to collagen-induced arthritis^4^, inducible alveolitis^5^, and spontaneous SLE^6^. The restoration of *FcγRIIB* expression using a retroviral vector prevents the SLE features of the phenotype^6–8^. Low expression of *FcγRIIB* has been observed in memory B cells, dendritic cells and plasmablasts in patients with SLE^9,10^.

*FcγRIIB* transmits its inhibitory signal via an immunoreceptor tyrosine-based inhibitory motif (ITIM) in its cytoplasmic domain^11^. The stimulation of ITIM-containing *FcγRIIB* and B cell receptor recruits phosphatases including SH2 domain containing inositol polyphosphate 5’ phosphatase (SHIP) and SH2 domain containing protein tyrosine phosphatase 1 (SHIP1). These result in the inhibition of several signaling cascades, including B cell receptor signaling via dephosphorylation of downstream targets. Patients with SLE have exacerbated periodontal disease severity caused by overproduction of proinflammatory cytokines including IL-6, IL-17, and IL-33^12^. In addition, high level of TNFα are seen and are correlated with both overall disease activity of SLE and periodontitis^13^. TNFα can augment osteoclastic bone resorption via its ability to enhance RANKL-driven osteoclast differentiation.

We recently demonstrated that mice lacking *FcγRIIB* displayed characteristic features of SLE-like disease such as inflammation-induced cortical and cancellous bone loss driven by increased bone resorption without alteration in bone formation in tibiae^14,15^. In addition, serum levels of TNFα were increased in *FcγRIIB*^*-/-*^ mice, and TNFα blockade ameliorated mandibular bone loss in this model^15,16^. However, while TNFα blockade is a promising approach to prevent SLE-associated mandibular bone loss, anti-TNFα biologics are associated with serious toxicities, including a greatly increased risk of mycobacterial and other infections and a potential increased risk for lymphoma or skin cancer development^17^. Anti-TNFα therapies can also be associated with paradoxical inflammatory responses, such as uveitis^18^. These limitations of anti-TNFα therapies justify a search for additional pathways mediating SLE associated mandibular bone loss to identify further druggable targets. To address this and identify new candidate mediators of SLE-associated mandibular bone loss in the *FcγRIIB*^*-/-*^ mouse model, we performed RNA-seq analysis to compare the gene expression profile of bone cells from mandibles in *FcγRIIB*^*-/-*^ mice and WT controls. The differentially expressed genes (DEGs) between groups were analyzed to evaluate the enriched pathways using the gene ontology database. Here, we demonstrated that 6-month-old *FcγRIIB*^*-/-*^ mice were osteopenic due to downregulation of osteoblast and osteoclast gene expression. RNA-seq analysis showed that suppressor of fused (*Sufu*) and serine protease inhibitor (*Serpina12*) gene expression were decreased and Hedgehog signaling was downregulated by *FcγRIIB* deficiency. siRNA-mediated knockdown of *Sufu* and *Serpina12* reduced osteoclast and osteoblast differentiation, respectively. Our studies demonstrate that *FcγRIIB* deficiency results in disruption of *Sufu* and *Serpina12* mRNA expression, leading to downregulation of Hedgehog signaling. This identifies the Hedgehog pathway as an unexpected candidate mediators of mandibular bone loss and justifies pharmacologic consideration of this pathway for SLE associated mandibular bone loss.

## Results

### Mice lacking *FcγRIIB* exhibit mandibular bone loss and develop anemia at 6 months of age

**Figure 1:**
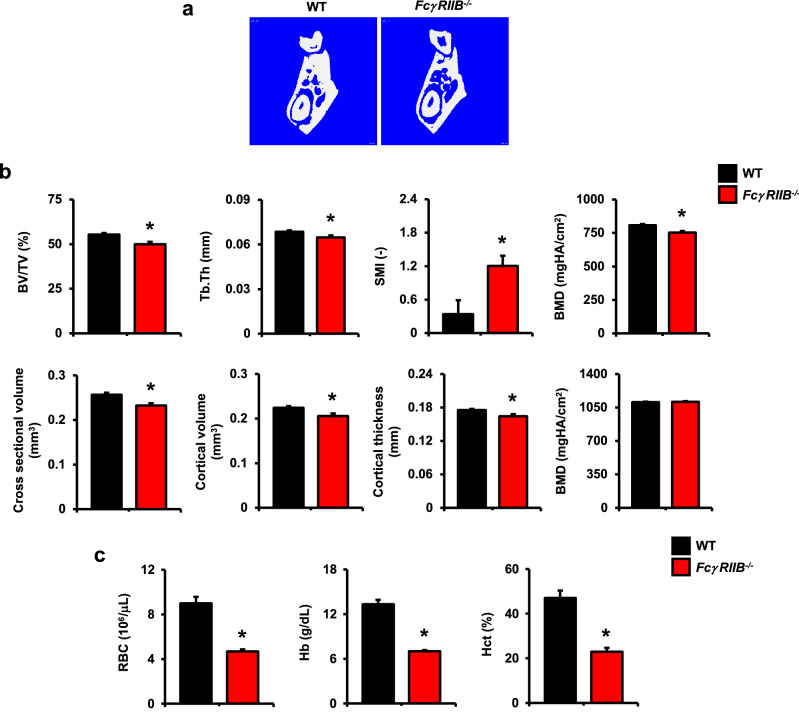
Ablation of *FcγRIIB* in mice induced mandibular bone loss and anemia. (**a**) Representative µCT images of the mandibles from 6-month-old *FcγRIIB*^*-/-*^ males compared to WT controls. (**b**) μCT analysis of cancellous bone (upper) and cortical bone (lower) at the first molar of the left mandible from *FcγRIIB*^*-/-*^ mice (*n* = 8) compared to WT controls (*n* = 6). (**c**) Complete blood count (CBC) analysis of 6-month-old *FcγRIIB*^*-/-*^ mice (*n* = 3) compared to WT controls (*n* = 4). Data are mean ± SEM. *p < 0.05 compared to WT controls. BV/TV; bone volume per tissue volume, Tb.Th; trabecular thickness, SMI; structural model index, BMD; bone mineral density, RBC; red blood cells, Hb; hemoglobin, and Hct; hematocrit.

To understand the basis of mandibular bone loss in the *FcγRIIB*^-/-^ mouse model of lupus, transcriptional profiling was performed. However, first we sought to confirm that *FcγRIIB* deletion caused mandibular bone loss in 6-month-old mice to validate our system. For this*,* µCT analysis was performed in accordance with suggested guidelines^19^. In line with our expectations^16^, cancellous bone volume, trabecular thickness, and bone mineral density (BMD) were significantly decreased in the mandibles of *FcγRIIB*^*-/-*^ mice compared to WT controls (Fig. 1a,b). The structural model index was increased in *FcγRIIB*^*-/-*^ mice, indicating a rod-like structure that is a characteristic of osteopenia. *FcγRIIB*^*-/-*^ mice had lower cross-sectional volume, cortical volume, and cortical thickness with no difference in cortical bone mineral density (Fig. 1b). *FcγRIIB* deficiency caused a perturbation in bone geometry as indicated by a decreased *Imin*, and *Imin/Cmin* although *J*, *Imax*, and *Imax/Cmax* did not change (data not shown). Thus, *FcγRIIB* deletion produces robust mandibular osteopenia, a feature also seen in human SLE.

To determine whether *FcγRIIB* deletion affected mandibular bone turnover, histomorphometric analysis was performed in 6-month-old *FcγRIIB*^**-/-**^ mice and WT controls. Consistent with µCT analysis, cancellous bone volume was decreased (Table 1). Although trabecular thickness was not altered, trabecular number was reduced with concomitant increase in trabecular separation. Osteoblast surface, osteoblast number, osteoclast surface, and osteoclast number were all decreased, indicating low bone turnover. Eroded surface was reduced. *FcγRIIB* deficiency caused hematopoietic defects similar to those associated with human SLE, with decreases in red blood cell counts, hemoglobin, and hematocrit in *FcγRIIB*^*-/-*^ mice (Fig. 1c). Consistent with an inflammatory or “anemia of chronic disease” etiology as opposed to impaired iron homeostasis, MCV, MCH, MCHC and RDW were not significantly altered (Supplementary Table S1). Thus, robust SLE features, including anemia and mandibular bone loss were observed in 6 months old *FcγRIIB*^*-/-*^ mice.

**Table 1:** Histomorphometric analysis of mandibles from 6-month-old *FcγRIIB*^-/-^ mice and their WT controls.

Parameters	WT	*FcγRIIB*^-/-^
	(n = 6)	(n = 6)
BV/TV (%)	55.42 ± 1.42	44.72 ± 2.07*
Tb.Th (μm)	74.61 ± 2.59	80.53 ± 4.07
Tb.N (/mm)	7.48 ± 0.35	5.59 ± 0.28*
Tb.Sp (μm)	60.69 ± 4.83	100.52 ± 7.41*
Ob.S/BS (%)	8.00 ± 0.42	6.45 ± 0.22*
N.Ob/T.Ar (/mm^2^)	102.59 ± 5.24	69.04 ± 4.72*
Oc.S/BS (%)	0.143 ± 0.018	0.067 ± 0.006*
N.Oc/T.Ar (/mm^2^)	1.22 ± 0.08	0.54 ± 0.03*
ES/BS (%)	0.212 ± 0.025	0.125 ± 0.013*

Results are mean ± SEM. *p < 0.05 compared to WT, unpaired *t*-test.

### RNA-seq reveals differential gene expression in skeletal cells from ***FcγRIIB***^-/-^ mice

**Figure 2:**
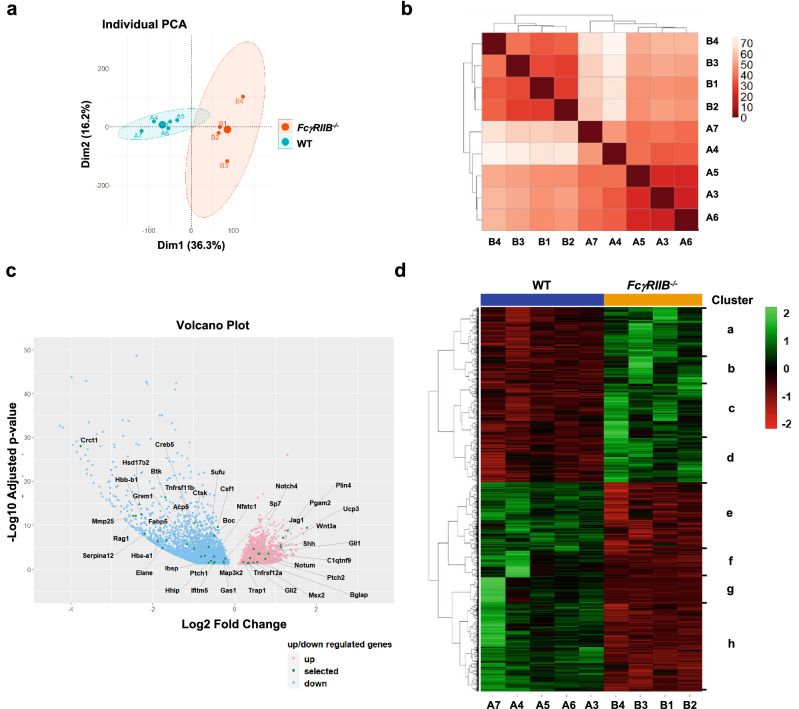
Transcriptional profiling identified differential gene expression in bone cells from WT and *FcγRIIB*^-/-^ mice. (**a**) PCA analysis of DEGs. The percentage variation for each dimension (Dim) are indicated in bracket on horizontal and vertical axis. (**b**) Sample-to-sample distance based on similarity of their gene expression profiles identified by Spearman’s rank correlation between samples. The shorter the distance, the more closely related the samples are. (**c**) Volcano plot of DEGs. Red dots represent significantly upregulated genes (*n* = 2,710) and blue dots represent significantly downregulated DEGs (*n* = 3,252) compared to WT controls. (**d**) Hierarchical clustering heatmap of DEGs across two comparisons. Green represents high gene expression and red represents low gene expression. Color descending from green to red indicates the z-score of log2 fold change (log2 FC) from high to low expression.

**Figure 3:**
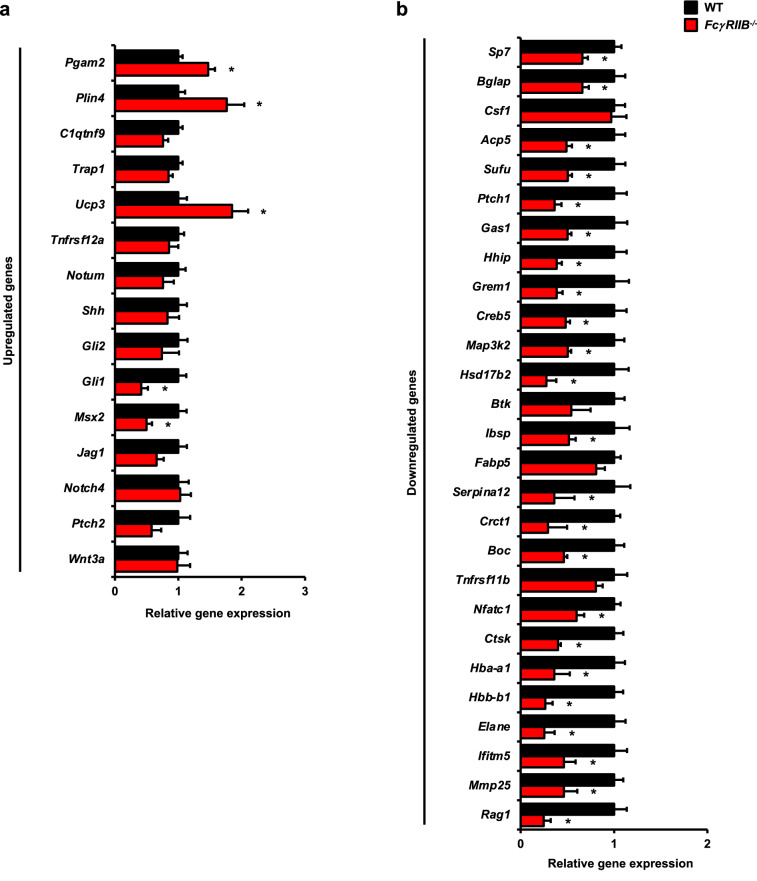
RNA-seq results were validated by qPCR analysis. (**a**) Upregulated and (**b**) Downregulated candidate genes in *FcγRIIB*^*-/-*^ mice (*n* = 6) compared to WT controls (*n* = 6). Data are mean ± SEM. *p < 0.05 compared to WT controls.

To better understand the mechanism by which deletion of *FcγRIIB* induced mandibular bone loss, RNA-seq was performed to identify DEGs in mandibular bone cells from 6-month-old *FcγRIIB*^-/-^ mice (*n* = 4) and WT controls (*n* = 5). RNA-seq analysis with a false discovery rate (FDR)-adjusted *p*-value of < 0.05 displayed 2,710 upregulated DEGs and 3,252 downregulated DEGs. Principal component analysis (PCA) conducted on all significant DEGs exhibited consistent separation based on genotype. PCA showed the sample relationship among specimens based on dimensions Dim1 and Dim2, which accounted for 36.3, and 16.2% of the variance, respectively (Fig. 2a). A correlation matrix heatmap represented the Euclidean distance between *FcγRIIB*^-/-^ mice and WT controls (Fig. 2b). Volcano plot analysis of DEGs showed both upregulated and downregulated genes in WT versus *FcγRIIB*^*-/-*^ mice (Fig. 2c). Hierarchical clustering heatmap analysis of DEGs identified in WT versus *FcγRIIB*^*-/-*^ mice demonstrated strong clustering within each of the two groups, showing 4 clusters for upregulated DEGs (a-d) and 4 clusters for downregulated DEGs (e–h) (Fig. 2d). Based on the RNA-seq data, 15 up and 27 downregulated DEGs were quantified by qPCR analysis. For upregulated gene identified by RNA-seq analysis, *Pgam2*, *Plin4*, and *Ucp3* expression were increased by qPCR analysis (Fig. 3a). Twenty three out of 27 downregulated genes identified by RNA-seq analysis such as *Sufu* and *Serpina12* were significantly decreased after validation (Fig. 3b). Approximately 62% (26/42) of validated genes revealed expression patterns similar to results from RNA-seq data. Several Hedgehog responsive genes were significantly dysregulated in *FcγRIIB*^-/-^ mice, including 13 genes, *Shh*, *Ptch2*, *Gli1*, *Gli2*, *Jag1*, *Creb5*, *Boc*, *Gas1*, *Hhip*, *Grem2*, *Ptch1*, *Hhipl1* and *Sufu* in 6-month-old *FcγRIIB*^-/-^ mandibles.

### Decreased expression of osteoclast and myeloid differentiation genes in ***FcγRIIB***^***-/-***^ mice

**Figure 4:**
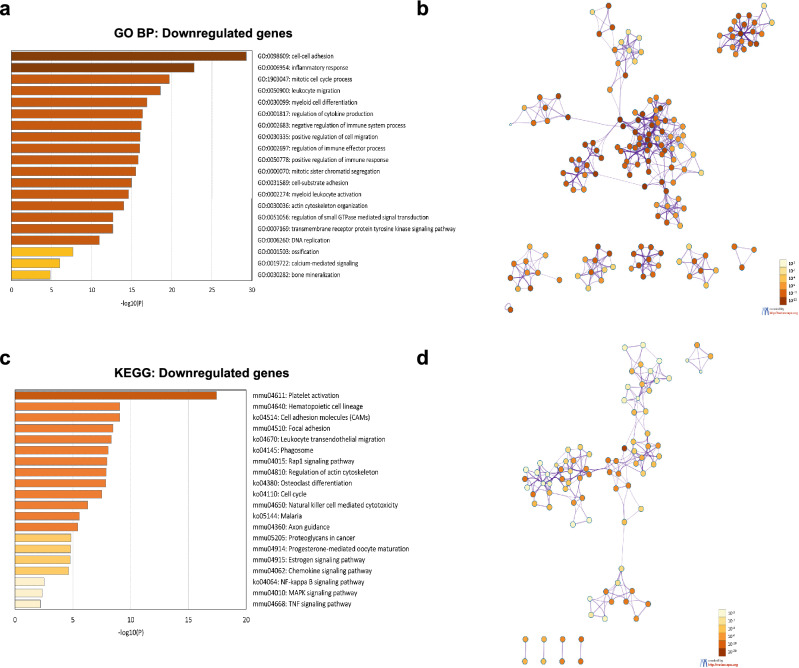
Enrichment analysis of the downregulated genes in *FcγRIIB*^-/-^ mice was performed using Metascape. (**a**) Bar graph showing top 20 enrichment clusters of GO terms. (**b**) The network of enriched GO biological processes (colored by *p*-values) (**c**) Bar graph showing top 20 enrichment clusters of KEGG pathways. (**d**) The network of enriched KEGG pathways (colored by *p*-values). The enriched GO biological processes and KEGG pathways were ranked by -log10 *p*-value of gene enrichment.

**Figure 5:**
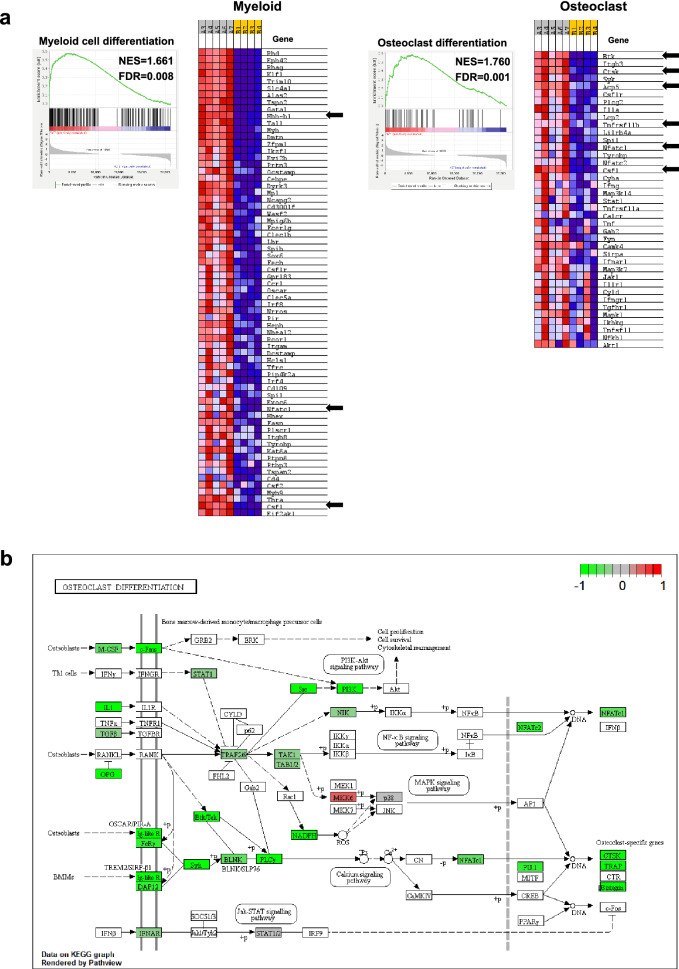
Downregulated genes in bone cells from *FcγRIIB*^*-/-*^ mice associated with osteoclast and myeloid cell differentiation. (**a**) GSEA identified downregulated DEGs in osteoclast (right) and myeloid cell differentiation (left). Heatmap indicating that osteoclast- and myeloid-related genes were differentially expressed in bone cells from *FcγRIIB*^*-/-*^ mice compared to WT controls with FDR-adjusted *p* < 0.05 as a cutoff. Red and blue represent up and downregulated genes, respectively. Arrows indicate 6 osteoclast and 3 myeloid genes that were validated using qPCR. NES; normalized enrichment score, and FDR; false discovery rate. (**b**) Downregulated DEGs are associated with osteoclast differentiation by KEGG pathway mapping.

To further identify which pathways may be perturbed in *FcγRIIB*^*-/-*^ mice, gene ontology (GO) and Kyoto encyclopedia of genes and genomes (KEGG) pathways were analyzed using Metascape. Among the biological processes represented by downregulated DEGs, the significantly enriched GO terms mainly consisted of cell–cell adhesion, inflammatory responses, mitotic cell cycle process, leukocyte migration, myeloid cell differentiation, regulation of cytokine production, negative regulation of immune system process, positive regulation of cell migration, regulation of immune effector process, positive regulation of immune response, mitotic sister chromatid segregation, cell-substrate adhesion, myeloid leukocyte activation, actin cytoskeleton organization, regulation of small GTPase mediated signal transduction, transmembrane receptor protein tyrosine kinase signaling pathway, DNA replication, ossification, calcium-mediated signaling, and bone mineralization (Fig. 4a,b). There were additionally several highly enriched KEGG pathways, including platelet activation, hematopoietic cell lineage, cell adhesion molecules (CAMs), focal adhesion, leukocyte transendothelial migration, phagosome, Rap1 signaling pathway, regulation of actin cytoskeleton, osteoclast differentiation, cell cycle, natural killer cell mediated cytotoxicity, malaria, axon guidance, proteoglycans in cancer, progesterone-mediated oocyte maturation, estrogen signaling pathway, chemokine signaling pathway, NF-kappa B signaling pathway, MAPK signaling pathway, and TNF signaling pathway (Fig. 4c,d). Many gene sets (FDR-adjusted *p*-value < 0.05) involved in bone resorption and development of myeloid lineage cells are enriched in these GO terms and KEGG pathways, such as osteoclast differentiation (*Btk, Ctsk*, *Acp5*, *Tnfrsf11b*, *Nfatc1*, *Csf1*) and myeloid cell differentiation (*Hbb-b1*, *Nfatc1*, *Csf1*) (Fig. 5a). KEGG pathway maps unveiled several downregulated genes involved in osteoclast differentiation (Fig. 5b)^20^.

### The Hedgehog signaling pathway contributes to skeletal changes in ***FcγRIIB***^-/-^ mice

**Figure 6:**
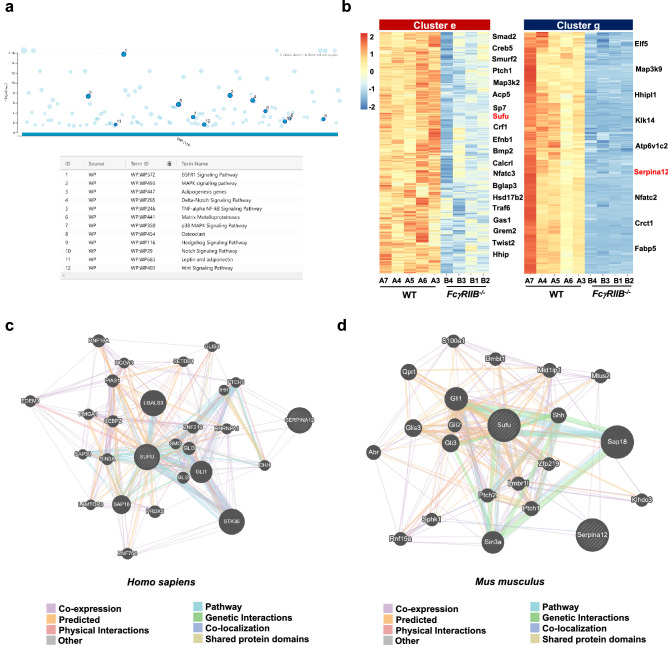
The Hedgehog signaling pathway enriched in downregulated DEGs may contribute to mandibular bone loss in *FcγRIIB*^-/-^ mice. (**a**) g:Profiler showing pathway enrichment in downregulated DEGs from the WikiPathways. The size of circle represents number of gene counts in each pathway. (**b**) Both *Sufu* and *Serpina12* were expressed in downregulated DEGs as shown in cluster e and g, respectively. (**c**) GeneMANIA network identifying potential interactions with *Sufu* and *Serpina12* associated with Hedgehog signaling pathway in human and (**d**) in mouse. The network was based on the genes retrieved from GeneMANIA by inputting *Sufu* and *Serpina12*.

g:Profiler, another tool for performing analysis based on WikiPathways, indicated that downregulated genes were associated with pathways regulating bone metabolism such as EGFR1 signaling pathway, MAPK signaling pathway, adipogenesis genes, Delta-Notch signaling pathway, TNFα NF-kB signaling pathway, matrix metalloproteinases, p38 MAPK signaling pathway, osteoclast, Hedgehog signaling pathway, Notch signaling pathway, Leptin and adiponectin, and the Wnt signaling pathway (Fig. 6a). Many downregulated DEGs were associated with Hedgehog signaling, particularly cluster e and g that may affect bone turnover in *FcγRIIB*^*-/-*^ mice. *Creb5, Ptch1, Sufu, Gas1, Grem2, Hhip,* and *Hhipl1* are Hedgehog responsive genes (Fig. 6b). *Serpina 12* known as vaspin, a visceral adipose tissue-derived serine protease inhibitor, has anti-inflammatory action to suppress the production of TNFα and proinflammatory adipokines^21^. The mRNA level of *Serpina12* was decreased in peripheral blood mononuclear cells of patients with SLE^22^. Thus, we focused on the DEGs (FDR-adjusted *p*-value < 0.01) that were downregulated in osteoclasts and osteoblasts, including *Sufu* and *Serpina12*, respectively.

The interactions of *Sufu* and *Serpina12* with other proteins were analyzed using GeneMANIA. The protein–protein interaction results indicated that *Sufu* and *Serpina12* showed 516 and 379 candidate interactions in humans and mice, respectively (Fig. 6c,d). Many proteins are associated with Hedgehog signaling and downstream targets of the pathway. In human, SUFU co-expressed with GLI1, DHH, STK36, ZNF219, and HMGA1 and co-localized with SMO, GLI1, and STK36 proteins (Fig. 6c). SERPINA12 co-expressed with SMO, PTCH1, IHH, and DHH proteins. In mouse, Sufu co-expressed with Lmbr1l, Ptch2, Gli2, and Mtus2, and co-localized with Gli3 and Zfp219 proteins (Fig. 6d). Serpina12 co-expressed with Gli1, Sphk1, and Mtus2 and co-localized with Lmbr1l and Sphk1 proteins. These results suggested that *Sufu* and *Serpina12* are novel candidate genes contributing to the osteopenic phenotype in mandibles of *FcγRIIB*-deficient mice and that Hedgehog signaling pathway is dysregulated in *FcγRIIB*^*-/-*^ mice.

### Targeted silencing of *Sufu* and *Serpina12* attenuates osteoclast and osteoblast differentiation

**Figure 7:**
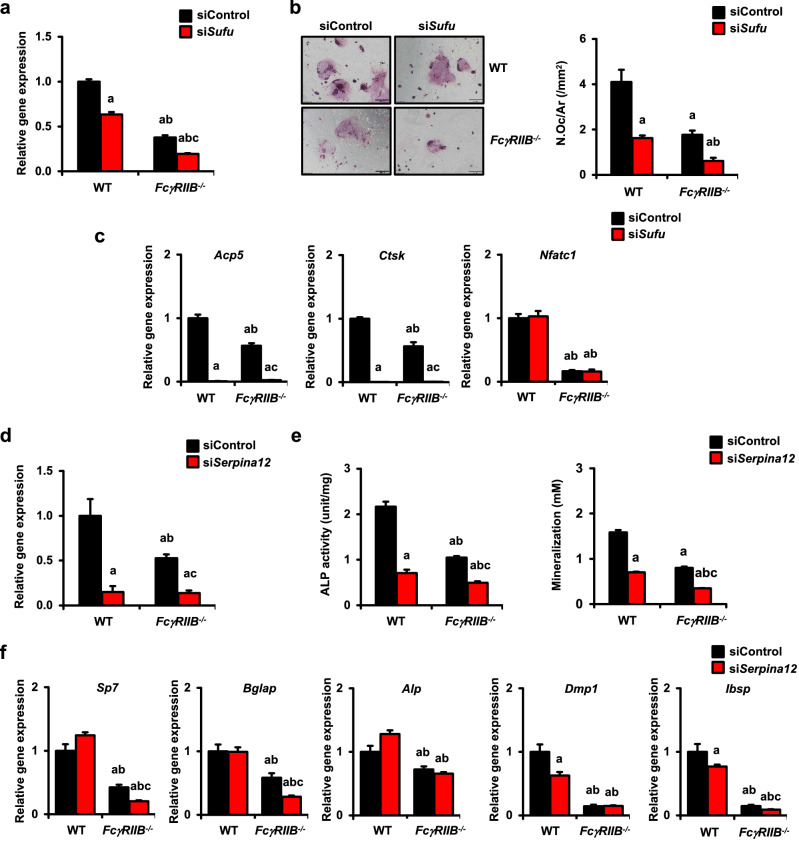
*Sufu* and *Serpina12* deletion impaired osteoclastogenesis and osteogenesis in bone cells from WT and *FcγRIIB*^*-/-*^ mice, respectively. (**a**) qPCR analysis of the efficiency of *Sufu* silencing in BMMs from mandibles of 6-month-old *FcγRIIB*^*-/-*^ males and WT controls (*n* = 3). (**b**) BMMs were transfected with si*Sufu* and siControl in the presence of M-CSF and RANKL. TRAP-positive osteoclasts with more than 5 nuclei were quantified using OsteoMeasure software. **c** The qPCR analysis of osteoclast gene expression after si*Sufu* transfection. (**d**) qPCR analysis of the efficiency of *Serpina12* silencing in osteoblasts from *FcγRIIB*^*-/-*^ mice and WT controls (*n* = 4). (**e**) Osteoblasts were transfected with si*Serpina12* and siControl in differentiation media. ALP activity (left) and mineralization (right) were quantified. (**f**) The qPCR analysis of osteoblast gene expression after si*Serpina12* transfection. Data are mean ± SEM. ^a^*p* < 0.05 compared to WT cells transfected with siControl, ^b^*p* < 0.05 compared to WT cells transfected with either si*Suf*u or si*Serpina12*, and ^c^*p* < 0.05 compared to *FcγRIIB*^*-/-*^ cells transfected with siControl. ALP; alkaline phosphatase, N.Oc; osteoclast number and Ar; area. Scale bar: 200 μm.

To investigate whether *Sufu* and *Serpina12* genes regulated bone homeostasis, we transfected BMMs and primary osteoblasts with siRNAs directed against *Sufu* and *Serpina*12, respectively. Consistent with the RNA-seq results, *Sufu* mRNA levels were significantly lower in *FcγRIIB*^-/-^ osteoclast precursor cells compared to WT controls (Fig. 7a). In addition, osteoclast number was decreased, indicating that *Sufu* negatively regulated osteoclastogenesis (Fig. 7b). Knockdown efficiency of *Sufu* using siRNA was confirmed by qPCR. *Sufu* expression was decreased by 37, and 62% in WT and *FcγRIIB*^*-/-*^ cells transfected with *Sufu* targeting siRNAs, respectively (Fig. 7a). After *Sufu* knockdown, the generation of TRAP-positive multinucleated osteoclasts was remarkedly decreased from both WT and *FcγRIIB*^*-/-*^ osteoclast precursors (Fig. 7b). The expression of osteoclast genes such as *Acp5*, *Ctsk* were also reduced in *Sufu*-knockdown cells (Fig. 7c). Knockdown efficiency of *Serpina12* was 90 and 68% in WT and *FcγRIIB*^*-/-*^ osteoblasts, respectively (Fig. 7d). ALP activity was decreased in *FcγRIIB*-deficient cells and *Serpina12* knockdown dramatically reduced ALP activity in both WT and *FcγRIIB*^*-/-*^ osteoblasts (Fig. 7e). Moreover, *Serpina12*-targeted siRNA also decreased mineralization in both WT and *FcγRIIB*^*-/-*^ osteoblasts as qualified by alizarin red staining (Fig. 7e). Consistently, the expression of osteoblast marker genes including *Sp7*, *Bglap*, and *Ibsp* was attenuated after *Serpina12* knockdown in *FcγRIIB*-deficient cells (Fig. 7f). Knockdown of *Serpina12* decreased *Dmp* and *Ibsp* expression in WT osteoblasts. These findings indicated that *Sufu* plays important role in osteoclast differentiation and that *Serpina12* is a key regulator of osteoblastogenesis that may contribute to the skeletal phenotype occurring in lupus.

## Discussion

SLE patients have at high risk of developing periodontitis, possibly due to increased salivary levels of pro-inflammatory cytokines^23^. The exact cause of SLE remains unknown, but accumulating evidence indicate that a combination of genetic susceptibility, cellular environment, and hormonal factors contribute to SLE development. MRL/*lpr*, NZB/W F1, and BXSB/MpJ-Yaa mice spontaneously develop lupus-like diseases characterized by an increase in anti-dsDNA antibodies, arthritis, splenomegaly, and glomerulonephritis^24^ similar to lupus patients. The low-affinity IgG receptor *FcγRIIB* contributes crosstalk between the skeletal and immune systems^25^. In contrast, deletion of the high-affinity Fc receptor *FcγRIV* was able to protect against inflammation-induced joint destruction in arthritic mice^26^. Imbalances in T helper cell (Th17) and regulatory T cells (Treg) in peripheral blood can contribute to SLE development or pathogenesis through increases in pro-inflammatory cytokines associated with active disease^27^. The inhibitory *FcγRIIB* combined with stimulatory *FcγRIIA* are potential regulators of periodontitis and SLE in patients^28^.

Our previous studies indicated that mice with *FcγRIIB* deficiency were osteopenic in tibiae at 6 months of age^14,15^. Similar to tibiae, cancellous bone volume and cortical bone volume were reduced in the mandible. 6-month-old *FcγRIIB*-deficient mice also develop anemia with a decrease in RBC number, hemoglobin, and hematocrit. Hematological abnormalities including anemia are found in SLE patients^29^. In line with this, decreased expression of hemoglobin genes linked with erythrogenesis (*Hbb-b1*, and *Hba-a1*) was observed.

Patients with SLE have a high risk of developing periodontitis, and periodontitis is a contributor to the multifactorial process of mandibular bone loss in SLE. The imbalance between host and microbiota in favor of a dysbiotic condition results in increased inflammatory response in periodontitis. These patients have elevated bacteria at periodontal disease sites, including *Prevotella oulorum and Prevotella pleuritidis, Pseudomonas spp, Treponema maltophilum, and Actinomyces IP073* compared to non-SLE controls^2^. The genetic variants in *FcγRIIB* change susceptibility to bacterial infection, and the *FcγRIIB* polymorphisms have been associated with periodontitis^30^. We do not observe histologic evidence of periodontal disease in *FcγRIIB*^-/-^ mice, and therefore aspects of bone loss related to periodontitis or oral dysbiosis are not necessarily captured in this model. However, it cannot be excluded that there is subclinical dysbiosis of the oral microflora that may contribute to bone loss in this model. Ultimately, addressing the role of the oral microflora in the changes observed here will be an important next step to advance these findings.

RNA-seq analysis is a powerful tool to investigate functional diversity and heterogeneity of cellular population. By comparative heatmap analysis and imperative validation of bone cells from *FcγRIIB*^*-/-*^ mice compared to WT controls, we identified 2,710 upregulated DEGs and 3,252 downregulated DEGs. Using qPCR validation, the expression of *Pgam2*, *Plin4*, and *Ucp3* were significantly increased in *FcγRIIB*^*-/-*^ mice. *FcγRIIB*^*-/-*^ mice had decreased gene expression related to myeloid cell differentiation (*Hbb-b1*, *Nfatc1*, *Csf1*, *Hba-a1*, and *Elane*), osteoclast function (*Ctsk*, and *Acp5*), and osteoclast differentiation (*Nfatc1*, *Tnfrsf11b*, *Csf1*, and *Btk*). *Btk* modulates B cell receptor (BCR) signaling and it acts downstream of the Fcγ receptor involved in osteoclast differentiation. Btk antagonists can improves the clinical features of lupus in the MRL/lpr, NZB/W F1, and BXSB/MpJ-Yaa mice as well as in pristane-induced DBA/1 lupus^31–33^. Based on these pre-clinical studies, the FDA approved Btk antagonist, ibrutinib, as a potential therapeutic agent for SLE patients.

Our results found that downregulated DEGs are associated with hedgehog signaling, particularly cluster e and g that may affect bone turnover in *FcγRIIB*^*-/-*^ mice. *Creb5, Ptch1, Gas1, Grem2, Hhip,* and *Hhipl1,* and *Sufu* are hedgehog responsive genes. Hedgehog signaling not only acts upstream within osteoblasts via the OPG/RANKL/RANK axis, but it is also involved in osteoblast differentiation. *Sufu* is expressed in bone marrow cells and has negative roles in regulating hedgehog signaling. *Sufu* deletion leads to failure of calvarial ossification, indicating a *Sufu* function in osteogenic lineage cells^34^. A decrease in the osteogenic regulators, *Runx2* and *Osx*, was found in calvarial mesenchyme of *Sufu* mutants. Deletion of *Sufu* stimulates hedgehog signaling in macrophage and inhibit RANKL-induced osteoclastogenesis^35^. Li et al. indicated that *Sufu* deletion in the dental mesenchyme using Dermo1-Cre induced delayed development of mandibular molars^36^. Multiple studies emphasized that *Serpina12* acts as a coordinator of the dynamic balance between bone formation and resorption. *Serpina12* attenuates osteoblast differentiation though PI3K‐Akt/miR‐34c mediated Runx2 transcriptional repression^37^. In contrast, Wang et al. revealed the protective effect of *Serpina12* in high fat diet (HFD)-induced bone loss in rats. In primary osteoblasts, osteogenic differentiation and ALP activity were increased after *Serpina12* treatment by increasing expression of *Runx2*, *Osx* and *Col1a1* and activating the Runx2 and Smad2/3 signaling pathways^38^. However, the mechanism of *Serpina12* on hedgehog signaling mediated bone turnover is still incompletely understood. In this report, we identified *Sufu* and *Serpina12* as candidate regulators of the hedgehog signaling pathway during lupus development. Additionally, we identified a link between *Sufu*, *Serpina12*, and overall hedgehog signaling and the mechanisms governing the expansion of mandibular bone loss in the *FcγRIIB*^*-/-*^ model of lupus.

The majority of downregulated DEGs were mapped within GO biological process, KEGG, and WikiPathways. They were involved in myeloid cell differentiation, ossification, calcium-mediated signaling, bone mineralization, and osteoclast differentiation. These data confirmed qPCR analysis that deletion of *FcγRIIB* decreased markers of osteoblast and osteoclast differentiation. In addition, *FcγRIIB* deficiency was associated with alterations in many signaling pathways including Hedgehog, EGFR1, MAPK, adipogenesis, Delta-Notch, TNFα NF-kB, p38 MAPK, Notch, leptin and adiponectin, and Wnt. The functions of two downregulated DEGs, *Sufu* and *Serpina12* were investigated. *Sufu* and *Serpina12* showed associated functional pathways that overlapped with downstream Hedgehog signaling proteins, including Gli1, Gli2, Gli3, Ptch2 in mice, and GLI1, SMO, IHH, DHH, and PTCH1 in humans.

*Sufu* is a negative downstream regulator of the Hedgehog signaling pathway by modulation of *Gli* transcription factors. Hedgehog signaling has both anabolic and catabolic functions in osteoblasts. Hedgehog ligands bind to their receptor, Ptch1, and release transmembrane protein Smo thereby activating transcription of Hedgehog target genes. Our knockdown studies indicated that silencing *Sufu* in BMMs dramatically attenuated osteoclast differentiation in both WT and *FcγRIIB*^-/-^ cells. Osteoclast marker genes, including *Acp*5, and *Ctsk* were reduced in BMMs transfected with si*Sufu*. These data confirmed previous reports that ablation of *Sufu* affected not only pre-osteogenic differentiation of the calvarial mesenchyme^34^, but also inhibited RANKL-induced osteoclast formation and osteoclast-specific gene expression^35^. *Msx2* is a downstream effector of the *Shh/Gli3* pathway in the limb^39^. Binding of *Sufu* to *Gli1* prevents the nuclear accumulation of *Gli1*. Based on qPCR validation, *Msx2* and *Gli1* showed the opposite results from RNA-seq possibly due to *Sufu* downregulation.

*Serpina12* is involved in the *Smad2*/*Runx*2 signaling pathway. It increased osteogenic differentiation and ALP activity and upregulated mRNA expression of *Runx2*, *Osx* and *Col1a1* and Runx2, and Smad2/3 protein levels^38^. Our study indicated that knockdown of *Serpina12* in osteoblasts suppressed osteoblast differentiation as indicated by decreased ALP activity and mineralization in both WT and *FcγRIIB*^*-/-*^ osteoblasts. Osteoblast marker genes such as *Sp7*, *Bglap*, and *Ibsp* were reduced in *FcγRIIB*^*-/-*^ osteoblasts transfected with si*Serpina12*. These data indicated that *Sufu* and *Serpina12* are novel candidate genes contributing to *FcγRIIB* deficiency induced osteopenia and, thereby, lupus associated mandibular bone loss. Liu et al. reported that *Serpina12* induces the expression of miR-34c, a regulator of osteoblast differentiation by directly targeting *Runx2*^37^. *Runx2* regulates *Wnt*, *Fgf*, *Pthlh*, and Hedgehog target genes, including *Gli1*, *Ptch1* and *Ihh* in cranial sutures of *Runx2*^+/-^ mice^40^. Therefore, it’s possible that *Serpina12* may contribute to osteogenesis by regulating Hedgehog signaling cascades.

In conclusion, this study was the first to apply an integrated RNA-seq analysis to explore novel candidate genes and mechanisms by which the *FcγRIIB*-deficient mouse model of lupus induces mandibular osteopenia. *Sufu* and *Serpina12* were identified as candidate molecular targets regulating osteoclastogenesis and osteogenesis through the Hedgehog signaling pathway.

## Materials and methods

### Mice

*FcγRIIB*^-/-^ mice on C57BL/6 background were obtained from Dr. Silvia Bolland (NIAID, NIH, Maryland, USA). Genomic DNA from tails was used for genotyping as previously described^14^. Mice were housed at the Faculty of Medicine, Chulalongkorn University and had free access to water and standard rodent chow (C.P. Mice Feed, Perfect Companion Group Co., Ltd., Thailand). All mouse laboratory procedures conformed to standards for the use of laboratory animals that were approved by the Institutional Animal Care and Use Committee (IACUC) at the Faculty of Medicine, Chulalongkorn University. This study followed the ARRIVE guidelines (Animal Research: Reporting In Vivo Experiments) for animal studies. Six-month-old *FcγRIIb*^-/-^ males and WT controls were used in this study. They were anesthetized with isoflurane and sacrificed by cervical dislocation.

### µCT analysis

After fixation in 10% NBF, mandibles were subjected to μCT analysis using a desktop μCT35, (Scanco Medical AG, Bassersdorf, Switzerland) in accordance with suggested guidelines^19^. Bucco-lingual cross slices of the first molar in the furcation zone between mesial and distal root were scanned at a voxel size of 7 μm, 70 kVp, 113 μA and 800 ms integration time using a threshold of 330. Parameters included bone volume fraction (BV/TV, %), trabecular number (Tb.N, /mm), trabecular thickness (Tb.Th, mm), trabecular separation (Tb.Sp, mm), cross sectional volume (mm^3^), cortical volume (mm^3^), cortical thickness (mm) and bone mineral density (mgHA/cm^2^).

### RNA-seq analysis

Whole mandibles including teeth of WT and *FcγRIIB*^-/-^ mice were pulverized in LN_2_ and total RNA was isolated and purified using Trizol (Invitrogen, Carlsbad, CA, USA) and an RNeasy Mini kit (Qiagen, Germantown, MD, USA), respectively according to the manufacturer’s instructions. The quality of isolated RNAs was validated by RNA integrity number (RIN) using Bioanalyzer (Agilent 2100, Santa Clara, CA, USA). Two hundred ng total RNA was subjected to mRNA sequencing library construction using NEBNext® Ultra™ Directional RNA Library preparation kit (New England Biolabs, Ipswich, MA, USA). Briefly, poly(A) mRNA was selected using magnetic oligo (dT) bead, and cDNA synthesis was performed. AMPure XP beads (Beckman Coulter Genomic, Beverly, MA, USA) were used to purify cDNA from the reaction mix. Subsequently, the cDNA libraries were ligated to Illumina adapters. Library quality and quantity assessments were performed using a Bioanalyzer and fluorometer (DeNovix, Wilmington, DE, USA). The final cDNA libraries were diluted to 10 nM, pooled and subjected to cluster generation and 75 bp single end sequencing on the Illumina NextSeq 500 sequencer (Illumina Inc, San Diego, CA, USA) at Omics Sciences and Bioinformatics Center, Bangkok, Thailand.

Quality control of raw sequence data was performed using FASTQC software. Subsequently, adaptor and low quality sequences were removed using Trim Galore software, then mapped to mouse genome reference (mm10) using HISAT v 02.1.0 with default parameters. The expression level of each gene was quantified using featureCounts software^41^. DEGs between WT and *FcγRIIB*^*-/-*^ groups were conducted using the DESeq2 package^42^. Significant genes were selected by false discovery rate (FDR)-adjusted *p*-value < 0.05 with the fold change above 1.0 defined as upregulation whereas the genes with the fold change below 0.5 considered as downregulation. The up and downregulated genes were selected for further analysis. RNA-seq data were deposited in NCBI Gene Expression Omnibus (GEO) and were accessible through GEO series accession number GSE179155.

### Bioinformatics tools

RNA-seq data analysis and visualization were performed in R software (https://www.R-project.org/) to generate the hierarchical clustering heatmaps, volcano plot, and PCA. DEGs were subjected to GO functional analysis under biological process categories and KEGG pathway maps using Metascape software to determine significantly enriched signaling pathways within the genes of interest. Gene Set Enrichment Analysis (GSEA) and g:Profiler were used for pathway enrichment analysis^43^. In addition, the relations between pathways, shared protein domains, and the co-localization and co-expression of either *Serpina12* or *Sufu* were assessed using GeneMANIA (http://genemania.org).

### siRNA transfection

Bone marrow macrophages (BMMs) and osteoblasts were transfected twice with Silencer Select *Sufu* and *Serpina*12 siRNA (Thermo Fisher Scientific, Waltham, MA, USA) using Lipofectamine 3000 (Invitrogen, Carlsbad, CA, USA) as per manufacturer’s instructions, respectively. The sequences of the Silencer Select *Sufu* siRNA were 5′–GACUGGAGAUUAACAGCAATT-3′ (sense) and 5′-UUGCUGUUAAUCUCCAGUCCC-3′ (antisense). The Silencer Select targeting *Serpina*12 was 5′–GGAUGAGAAAGGCAUGGAATT-3′ (sense) and 5′-UUCCAUGCCUUUCUCAUCCAT-3′ (antisense). The Silencer Select Negative Control No. 1 siRNA (Thermo Scientific, Waltham, MA, USA) was used as a negative control. The efficiency of gene knockdown was validated by qPCR analysis and *Gapdh* expression was used as an internal control.

### Osteoclast culture

Bone marrow cells were flushed out from mandibles with α-MEM medium and cultured in α-MEM medium containing 10% FBS, 100 units/ml penicillin, and 100 ug/ml streptomycin overnight. Non-adherent cells were cultured in the same medium containing 20 ng/ml M-CSF for 24 h to generate BMMs. BMMs were used for transfection, osteoclast differentiation, and qPCR analysis. Transfected BMMs were cultured in the same medium containing 20 ng/ml M-CSF, and 3.3 ng/ml RANKL (R&D Systems, Inc., MN, USA). TRAP-positive osteoclasts containing more than 5 nuclei were quantified using OsteoMeasure software. Osteoclast number per total area (N.Oc/Ar) was analyzed. Osteoclasts were also kept at −80 °C for RNA isolation and qPCR analysis.

### Osteoblast culture

Mandibles were minced and digested by collagenase type II (Worthington Biochemical Corporation, Lakewood, NJ, USA) for 2 h at 37 °C. The bone fragments were cultured in α-MEM medium containing 20% FBS, 100 units/ml penicillin and 100 ug/ml streptomycin. Osteoblasts were plated in the same media for transfection, osteogenic differentiation, and qPCR analysis. Transfected osteoblasts were differentiated in media containing 5 mM β-glycerophosphate, 50 μg/ml ascorbic acid, and 10 μM dexamethasone. Alkaline phosphatase (ALP), and mineralization were quantified on day 7 and 21, respectively. Osteoblasts were kept at −80 °C for RNA isolation and qPCR analysis.

### qPCR analysis

The first strand cDNA synthesis was synthesized using SuperScript VILO kit (Invitrogen, Carlsbad, CA, USA). The qPCR was performed in Luna Universal qPCR master mix (New England Biolabs, Ipswich, MA, USA) using CFX96™ Optics Module (Bio-Rad, CA, USA). Relative gene expression was quantified and *Gapdh* was used as internal control. All primer sequences are listed in Supplementary Table S2.

### Histomorphometry

Mandibles were decalcified in 10% EDTA with daily changes at pH 7.3 for 3 weeks. Decalcified mandibles were dehydrated with ascending graded alcohol, and xylene, and embedded in paraffin. Samples were sectioned at a thickness of 5 µm using a Leica RM2255 microtome (Leica Biosystems Nussloch GmbH, Germany). Sections were deparaffinized and stained with haematoxylin and eosin (H&E). Cancellous bone volume per tissue volume (BV/TV, %), trabecular thickness (Tb.Th, μm), trabecular separation (Tb.Sp, μm), and trabecular number (Tb.N, /mm) were analyzed. Osteoblast surface per bone surface (Ob.S/BS, %), osteoblast number per tissue area (N.Ob/T.Ar, mm^2^), osteoclast surface per bone surface (Oc.S/BS, %), osteoclast number per tissue area (N.Oc/T.Ar, mm^2^), and eroded surface per bone surface (ES/BS; %) were quantified. Histomorphometric measurements were carried out using the OsteoMeasure system (OsteoMetric Inc.) according to standardized nomenclature^44^.

### Hematological analysis

Blood samples were collected from the retro-orbital sinus of 6-month-old *FcγRIIB*^*-/-*^ mice and WT controls. A complete blood count (CBC) was analyzed using automated hematology analyzer at Faculty of Veterinary Science, Chulalongkorn University.

### Statistical analysis

All statistical analyses were performed using SPSS 22 (IBM, Armonk, NY, USA). Student's t-test and one-way ANOVA followed by Fisher's protected least significant difference test were used to compare the differences between two groups and more than two groups, respectively. Data are expressed as mean ± SEM. *p* < 0.05 were considered statistically significant.

## Supplementary Information

Supplementary Information.

## Data Availability

All data generated for this study are available from the corresponding authors upon request.
