# Supplementary material for: Identification of candidate regulators of mandibular bone loss in FcγRIIB-/- Mice
Source: Sci Rep. 2021 Sep 21;11:18726. doi: 10.1038/s41598-021-98108-3 (PMC8455620; doi:10.1038/s41598-021-98108-3)
Supplement: Supplementary file 1 — Supplementary Information. [file 41598_2021_98108_MOESM1_ESM.pdf]

Identification of Candidate Regulators of Mandibular Bone Loss in *FcγRIIB*$^{-/-}$ Mice

Nithidol Sakunrangsit[1], Jatuphol Pholtaisong[2], Jeerus Sucharitakul[1,3], Sasithorn Wanna-udom[1], Pinidphon Prombutara[4], Prapaporn Pisitkun[5], Asada Leelahavanichkul[6], Chatchawit Aporntewan[7], Matthew B. Greenblatt[8], Sutada Lotinun[1,*]

[1]Skeletal Disorders Research Unit, Department of Physiology, Faculty of Dentistry, Chulalongkorn University, Bangkok 10330, Thailand
[2]Program in Bioinformatics and Computational Biology, Graduate School, Chulalongkorn University, Bangkok 10330, Thailand
[3]Department of Biochemistry, Faculty of Dentistry, Chulalongkorn University, Bangkok 10330, Thailand.
[4]Omics Sciences and Bioinformatics Center, Faculty of Science, Chulalongkorn University, Bangkok 10330, Thailand
[5]Division of Allergy, Immunology, and Rheumatology, Department of Medicine, Faculty of Medicine, Ramathibodi Hospital, Mahidol University, Bangkok 10400, Thailand
[6]Division of Immunology, Department of Microbiology, Faculty of Medicine, Chulalongkorn University, Bangkok 10330, Thailand
[7]Department of Mathematics and Computer Science, Faculty of Science, Chulalongkorn University, Bangkok 10330, Thailand
[8]Department of Pathology and Laboratory Medicine, Weill Cornell Medicine, and Research Division, Hospital for Special Surgery, New York, NY 10065, USA

*Address correspondence to:
Sutada Lotinun, Ph.D.
Department of Physiology
Faculty of Dentistry
Chulalongkorn University
Bangkok 10330, Thailand
Tel: +662 218-8694
Fax: +662 218-8691
Email: sutada.l@chula.ac.th

Short title: RNA sequencing: *FcγRIIB* deficiency induced mandibular bone loss in mice

Key words: Transcription factors, systemic lupus erythematosus, osteoblast, osteoclast, siRNA, periodontitis

Table S1 Hematological analysis of 6-month-old *FcγRIIb*$^{-/-}$ males and their control littermates.

Parameters	6-month-old	
	WT (n=4)	*FcγRIIb*$^{-/-}$ (n=3)
WBC (10^3/μL)	6.280±1.272	3.467±0.393
Neu (10^3/μL)	0.898±0.227	0.707±0.085
Lym (10^3/μL)	4.638±0.942	2.350±0.556
Mon (10^3/μL)	0.173±0.037	0.300±0.189
Eos (10^3/μL)	0.565±0.195	0.097±0.019
Bas (10^3/μL)	0.008±0.003	0.013±0.003
Neu (%)	14.20±2.68	20.83±3.61
Lym (%)	74.15±3.15	66.20±11.23
Mon (%)	3.075±0.749	9.633±6.775
Eos (%)	8.450±1.796	2.967±0.745
Bas (%)	0.125±0.048	0.367±0.120
RBC (10^6/μL)	8.985±0.580	4.667±0.191*
HGB (g/dL)	13.33±0.59	7.00±0.15*
HCT (%)	46.98±3.39	22.97±1.74*
MCV (fL)	52.75±4.60	49.60±5.44
MCH (pg)	14.90±0.34	15.10±0.35
MCHC (g/dL)	28.78±2.06	30.93±2.49
RDW-CV (%)	17.00±1.44	15.77±1.56
RDW-SD (fL)	41.50±7.11	35.43±7.32
PLT (10^3/μL)	942±49	601±200
MPV (fL)	5.500±0.183	6.167±0.186
PDW (-)	15.78±0.36	15.50±0.12
PCT (%)	0.523±0.040	0.362±0.110

$^{*}p<0.05$ versus WT controls, unpaired t-test.

Table S2 Oligonucleotide primers used for qPCR analysis.

Gene	Forward sequences	Reverse sequences
Ucp3	ACCCGATACATGAACGCTCC	TCATCACGTTCCAAGCTCCC
Pgam2	TGAACCTGCCCACTGGAATC	TCGTCTCCCAGGAACCTCAT
Plin4	ACTCCTGCCCCCTCATCTAA	CTTCGTATTGGTGAGGACATTCT
Wnt3a	TGGCAGAATGAGGCATGGAG	TCAGGAAAGCTCTGCCAAGG
Notum	ATTGCCATGGGAGGAGAGGT	AAAGCTGTCCATGTTGCCCT
Trap1	TTCACCTTCAGACGGATGCC	CTGGTCTCTGCCTGGTTCTG
C1qtnf9	GATTTGGTGGCTTCTGCTGG	TGTTTCCCCGGAGATCCTCT
Tnfrsf12a	TTGGGATTCGGCTTGGTGTT	GTCCATGCACTTGTCGAGGT
Rag1	GGCGGTCCAATCCGTATCAT	GGCCTGCCATCTCTTCCTTT
Mmp25	TAGACTGAGACGGCGGAGTA	AACGGCGATAATCGAGGGTC
Crct1	GTCTTTGCTGCTCTCACCCA	CTGCCCATAGCGTGATCTGA
Hsd17b2	AGTGGAGAATGAGCCCGTTT	GGAATCAGCACCTGTCACCA
Serpina12	ACAGCCACATTTGTCCTTCC	ACCTTCAGGCTTCGATGAGA
Nfatc1	AGGCTGGTCTTCCGAGTTCA	ACCGCTGGGAACACTCGAT
Acp5 (TRAP)	GATCCCTCTGTGCGACATCA	CCAGGGAGTCCTCAGATCCA
Tnfrsf11b (OPG)	AAGAGCAAACCTTCCAGCTGC	CACGCTGCTTTCACAGAGGTC
Alp	CTTGACTGTGGTTACTGCTGATCA	GTATCCACCGAATGTGAAAACGT
Sp7 (Osx)	CCCTTCTCAAGCACCAATGG	AAGGGTGGGTAGTCATTTGCATA
Bglap (Ocn)	GCTGCCCTAAAGCCAAACTCT	AGAGGACAGGGAGGATCAAGTTC
Dmp1	ATGACTGTCAGGACGGCTAC	AGTTATAGTGAACTCTCTAC
Ibsp (Bsp)	TGGCGACACTTACCGAGCTT	CCATGCCCCTTGTAGTAGCTGTA
Csf1	ACCTGTTTCCCAAGAAGAGAGCCT	AGCTGTCAACACAAGCAGCCAAAG
Msx2	ACTCTAACGTGTTGGGCAGA	TGGACAGGAAGGTGAGACAC
Shh	TCCGATGTGTTCCGTTACCA	CCTGAGGACTTGTGAGCTGT
Gli1	ACCCGGGATACAACCCAAAT	AGACCATTGCCCATCACAGA
Gli2	CTGCTGGCCTGGTGTCCTTA	TGGGGCAGAAGTCTCCATCT
Jag1	AAGAATGGCTCCAGGGTGAG	GACAACTGTGCCTGCTTAGG
Notch4	AGAGCTTCTGTGTGGAGGAT	CAGAGCTCTTCCAGATGGGT
Sufu	GTGCTATTGCCTTCCAAGCG	GCAGTGGGCTTTACCCTCTT

Map3k2	GAAGCAGACATGTGAAGCCA	ACTCCAAGACCCCAAGAACA
Creb5	CACCCTCAGTCAGCTTACAA	AAGGGTTGGGCTGGTAAGAA
Hba-a1	TGATGTAAGCCACGGCTCTG	GGTACAGGTGCAAGGGAGAG
Hbb-b1	GTCTCTTGCCTGTGGGGAAA	AAGGGTAGACAACCAGCAGC
Elane	ACCCAGTGTGCTACAAGAGC	GTGCATACGTTCACACGACG
Btk	AGGCCACAGGAGACCTTAAA	AATGCAGGTAGCTCCCCAGA
Ptch1	AGGCGCTAATGTTCTGACCA	CCTCCTGCCAATGCATATAC
Ptch2	TGGCTTCTCCCACAAGTTCA	CAGGAAGGTGCTCTGCAAGG
Ifitm5	CTGGTCTGTCTTCAGCACGA	CCGCAGAGTCTTTGGCTAAC
Gas1	ATGTTCGGCCCTCTTCTGTG	CTTGAAAGACCCCCACCGTT
Hhip	TCGAAACGGCTACTACACCC	CTCGAACTGTCCCAGAAACT
Grem1	GTCAAAGCGGGCACATTCAG	GGATGTTTGGGTCCAGTGGT
Boc	GACGGCGGTATCCCTACTTG	GTTCCTCCCAGCTTCTGGAC
Gapdh	TGCACCACCAACTGCTTAG	GGATGCAGGGATGATGTTC
